# Promoting bone callus formation by taking advantage of the time-dependent fracture gap strain modulation

**DOI:** 10.3389/fsurg.2024.1376441

**Published:** 2024-05-02

**Authors:** Dirk Wähnert, Marco Miersbach, Christian Colcuc, Stefano Brianza, Thomas Vordemvenne, Michael Plecko, Angelika Schwarz

**Affiliations:** ^1^ Department of Trauma and Orthopedic Surgery, Protestant Hospital of Bethel Foundation, University Hospital OWL of Bielefeld University, Bielefeld, Germany; ^2^ Biomech Innovations AG, Nidau, Switzerland; ^3^ Department of Orthopaedics and Traumatology, Trauma Hospital Graz (UKH), Graz, Austria

**Keywords:** secondary bone healing, dynamization, variable fixation locking screw, callus formation, fracture healing

## Abstract

Delayed union and non-union of fractures continue to be a major problem in trauma and orthopedic surgery. These cases are challenging for the surgeon. In addition, these patients suffer from multiple surgeries, pain and disability. Furthermore, these cases are a major burden on healthcare systems. The scientific community widely agrees that the stability of fixation plays a crucial role in determining the outcome of osteosynthesis. The extent of stabilization affects factors like fracture gap strain and fluid flow, which, in turn, influence the regenerative processes positively or negatively. Nonetheless, a growing body of literature suggests that during the fracture healing process, there exists a critical time frame where intervention can stimulate the bone's return to its original form and function. This article provides a summary of existing evidence in the literature regarding the impact of different levels of fixation stability on the strain experienced by newly forming tissues. We will also discuss the timing and nature of this “window of opportunity” and explore how current knowledge is driving the development of new technologies with design enhancements rooted in mechanobiological principles.

## 1 Introduction

Bone is an organ with the rare ability to regenerate without scar formation, allowing complete restoration of form and function. Despite all the promising new technologies to improve fracture care that have been introduced over the past decades from basic, translational and clinical research, 5%–10% of all fractures still fail to heal successfully or heal with delay (1). In addition, global population growth and aging have led to a significant 30% increase in the total number of fractures occurring worldwide between 1990 and 2018 (2). With different gender related age-specific fracture incidence, the demographic shift linked to longer lives and family size shrink has determined a substantial increase in the amount of fractures in older individuals (2). These osteoporotic fractures are associated with a high risk of complications. In addition to the complications associated with reduced bone strength (implant loosening and construct failure), these patients also have reduced biological potential for regeneration, often resulting in delayed fracture healing. As a result, osteoporotic fractures are a major source of suffering for these patients and a dramatic socioeconomic burden for society (1). Fractures occurring in the working-age population are related to considerable direct expenses and a substantial loss in productivity (3). Projections suggest that the cost and the societal burden of complicated fracture cases will become increasingly unsustainable in the upcoming years (4–11). Addressing this societal challenge necessitates an interdisciplinary and collaborative approach to assist policymakers in determining how to maximize the effectiveness of fracture prevention and treatment programs. From our side, we can contribute by enhancing our understanding of the latter.

The surgical community seems to agree that about 90%–95% of all osteosynthesis lead to restoration of the form and function of the affected bone at the first treatment (12–16). This success rate sounds promising if the disruption of the healing process leading to a complication and its associated consequences was not a catastrophic event. In fact, a healing complication resembles a “black swan” event in osteosynthesis—a highly unpredictable event that can have a significant impact on both the patient and the healthcare system.

For the patient, healing complications means a much longer treatment. Complications expose the patient to the risk of any other sort of further complications, such as hardware failure and infection (17). Furthermore, the long-term patient-related quality of life is lower in patients who experienced complications in comparison to normative data (18). To the healthcare system, the treatment of such minority of patients represents variable and often exorbitant costs (19). In working aged population the 5% experiencing poor healing has been estimated to more than double the work losses and medical costs (3).

Treatment duration significantly affects the cost and societal burden of fractures. Complicated cases have much longer treatment times, impacting the overall population healing time. Our contribution is thus not to be sought in trying to accelerate the fracture healing process in each patient but rather in increasing the chances to trigger and complete it in all patients and thus decreasing the number of complicated cases.

Although surgeons have identified potential risk factors that may contribute to unsuccessful fracture healing (20, 21) it is evident that patients with identical injury profiles and similar risk factors have different outcomes. Some experience successful healing and others do not (20). Identifying patients at a higher risk of developing non-union remains a persistent challenge for both clinicians and scientists (15). Monitoring the healing process using measurements that indicate fracture consolidation is a recent advancement. Continuous load measurement with smart implants allows early detection of healing abnormalities compared to traditional radiographic monitoring (22–24). Nevertheless, by the time a healing disturbance becomes detectable, the healing process is already significantly compromised.

Thus, if healing disturbances cannot be certainly predicted and identifying them once occurred is sometimes too late for an effective cure (15, 18), fostering fracture healing in all patients is the best prevention surgeons can consistently stage because it maximizes the chances for early segment restoration.

There is consensus in the scientific community about the stability of the fixation having a role in determining the fate of an osteosynthesis. The degree of stabilization determines fracture gap strain and fluid flow and triggers a positive or negative reaction of those processes contributing to the regenerative pattern (25–27). Several mechanical signals are induced in bone tissue during loading. These include stress, strain, fluid flow, and streaming potentials. Of these, the shear stress induced by interstitial fluid flow appears to be the most relevant signal for mechanotransduction (28, 29). Mechanically induced deformation has also been shown to increase oxygen transport, thereby improving nutrient delivery to cells involved in fracture repair (28, 30). Several concepts for implantable devices have been developed to stimulate the formation of bone callus through mechanobiological principles. Among those following the plating principles we mention both screws (31–39), plates (40–43) and surgical techniques (44) solutions. Most of these solutions aim at building constructs that are flexible or very flexible at low loads and that become abruptly rigid or very rigid by means of a contact mechanism. Designed to provide an immediate stimulus and avoid callus overstimulation, during the entire fracture healing process these solutions provide to the cells building the healing tissue a fairly constant stimulus according to patient loading. However, there is growing evidence in the literature that different phases of fracture healing profit from different level of stability. There seems to exist a window of opportunity be exploited to provide those stimuli increasing the chances to gain full return of form and function of the injured bone. A better understanding of the mechanical regulation of the fracture healing process is crucial for the development of new generations of devices and treatments. This manuscript summarizes the evidence available in the literature on the effect of timing and tissue strain on the formation and maturation of bone callus. We will speculate on the timing and strain range of the window of opportunity and report on how the current knowledge is fostering the development of new technologies featuring designs improvement based on pure mechanobiological concepts.

## 2 Is there a window of opportunity for fracture healing and what do we know about it today?

Secondary bone healing, is the form of bone healing most commonly associated with fractures that are prone to healing complications. It occurs through a cascade (15) of processes in response to fractures, with each step activating the next, triggering the sequential deposition of different tissues, and ultimately restoring the bone to its original form and function. The fracture of the bone disrupts the local vascularity and triggers the inflammatory cascade. During this first, inflammatory phase, the secretion of mediators into the fracture hematoma regulates the differentiation of mesenchymal stem cells into osteoblasts, fibroblasts and chondrocytes as well as cell infiltration and angiogenesis (45). In the subsequent fibrovascular phase, the non-perfused bone fragments are resorbed. The hematoma is replaced by the so-called bone callus, a granulation tissue mainly characterized by fibroblasts, further collagen and capillaries. In the following bone formation phase, mineral is disorganisedly deposited on the extracellular matrix, the fracture gap is bridged with solid material and the fractured bone regains its mechanical competence. In the last remodelling phase, the woven bone is replaced by organized lamellar bone and the macroscopic bone geometry is fully restored.

Among the various factors influencing the secondary fracture healing process, those related to the patient's metabolic status, comorbidities (46, 47), especially when combined (48, 49), open fracture (50), and the grade of tissues damage (51–53) have a large effect but cannot be controlled at surgery (54). While established habits, such as smoking, steroids, drugs and alcohol are known to have negative effect on fracture healing (15, 55, 56). Those related to fixation stability and surgical technique have been shown to have a positive or negative effect (49, 54, 57) and led to the development of different fixation techniques. Among these, the concept of biological osteosynthesis is in continuous development (16, 25, 26, 28, 45, 57–59). Despite angiogenesis and vascularization are essential for callus formation and maturation, it has been suggested (60) that promotion of fracture healing should focus on the stimulation of osteogenesis rather than on the stimulation of angiogenesis. In fact, it has been shown that extensive angiogenesis and vascularization may not support, but paradoxically hinder, adequate fracture repair and, thus increase the risk of non-union development (60). Studies have proven that stimulation of blood vessel formation did not determined a significant increase in bone formation (61–66) and the effect of surgical angiogenesis by the generation of arteriovenous bundles and vascularized bone grafts did not report an improved bone viability and union rate (67–69). Conversely, today it is well-accepted that techniques of internal fixation require some degree of motion at the fracture gap while keeping the contact between implant and bone stable (70).

**Figure 1:**
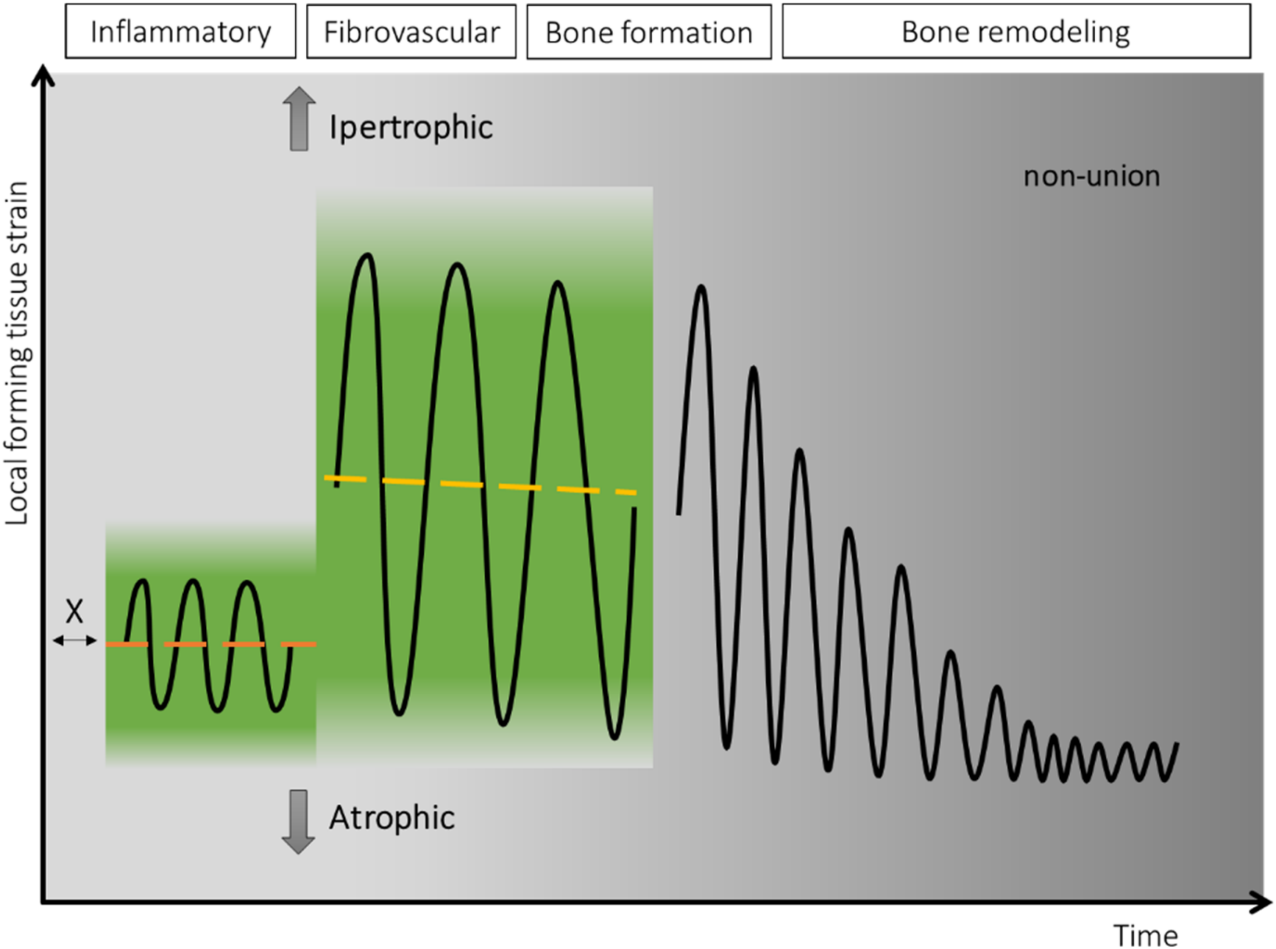
In green, the theoretical strain-temporal “window of opportunity” for fracture healing. On the *x*-axis the temporal dimension with the, in reality, partially superimposed four phases. Its temporal beginning (X) seems to be “injury-patient tailored (71)” while its extension is limited to the onset of the bone formation phase. On the *y*-axis the local forming tissue strain. In black, the schematic strain cycles represent the optimal deformation experienced by the newly formed tissue and transmitted to the embedded cells leading to restoration of the bone form and function.

Recent findings on the mechanobiology of fracture healing have motivated us to define a “window of opportunity” for the time-dependent mechanical requirements for successful fracture healing. This provides opportunities for the development and introduction of fixation techniques that best meet the needs of the different phases. Tissues are built by cells, and to optimize their osteogenetic capacity, we strive to understand the fracture healing process from the perspective of these cells. The window of opportunity is a strain-temporal environment where the fracture healing cascade can be triggered and completed (Figure 1). Outside the window of opportunity the cascade is not triggered or irretrievably compromised. After trauma, its temporal beginning seems to be injury-patient tailored (71), with local (MuST—Musculo—Skeletal Temporary surgery) vs. systemic (polytrauma: DCO—Damage Control Orthopedics) scenarios (72) profiting from different temporal and staged strategies (71, 72). Despite late dynamization has been proven to have a beneficial effect on the maturation of the bone callus (73, 74) the window of opportunity is confined to the sequential processes that initiate callus formation, stimulate its growth, and ultimately result in the formation of a robust bony bridge. This primarily occurs during the transition from the inflammatory phase to the early bone formation phase.

**Figure 2:**
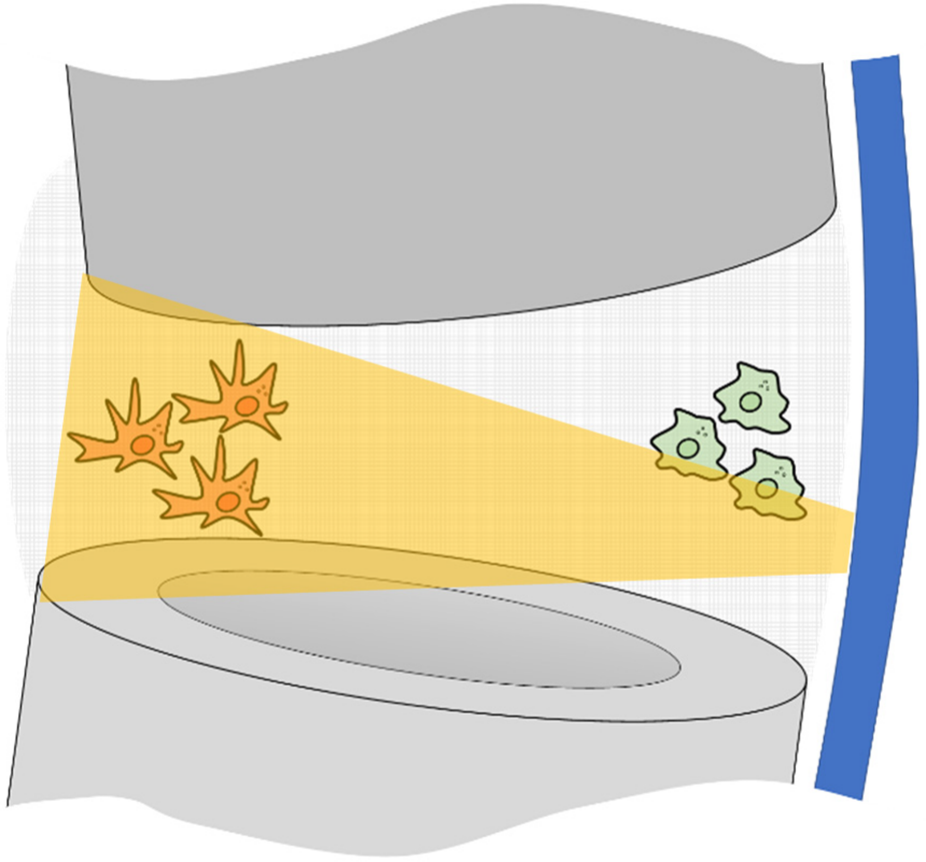
Pictorial representation of a fracture gap fixed with bridge plating. In grey, the proximal and distal bone segments; in blue the locking plate. The bone forming cells are affected by the strain of the surrounding tissue they are attached to. This depends on local gap size, on the loading applied to the affected bone, on the stiffness of the bone-implant construct, on the position with respect to the implant and on the changing mechanical properties of this same local forming tissue. Clinically this results in a substantially higher strain (orange trapezoid) provided to cells far from the plate (in red) and low strain for those close to the plate (in green).

The second dimension of the window of opportunity is the “local forming tissue strain”. The forming tissue strain is the local tissue deformation of the growing tissue during each healing phase. Acting on the tissue embedded cells and on the fluids flow, it is a local phenomenon promoting or inhibiting the healing cascade and locally leading to tissues growth and maturation. The local nature is evident with certain plate and screw bridging configurations where the interfragmentary strain is substantially different between the cis and trans cortex (75). The local forming tissue strain depends on the local gap size, on the loading applied to the affected bone, on the stiffness of the bone-implant construct (28), on the position with respect to the implant and on the mechanical properties of this same local forming tissue (Figure 2). Along the window of opportunity, the combination of all these factors creates a continuously changing local forming tissue strain that leads to the local formation, remodelling and resorption of the tissues' characteristic of each fracture healing phase.

Despite fracture healing phases overlap and often coexist, there is increasing evidence suggesting that the suitable strain conditions are not the same between fracture healing phases and that the early phases of fracture healing determine the fate of the fixation. At the beginning of the treatment, a relatively stable mechanical environment (76–79) has been proved to fosters blood vessels formation (80, 81) and the differentiation of mesenchymal cells towards an osteogenic rather than a chondrogenic lineage (27, 82, 83). A constant less-stable fixation slows down the transition from the inflammatory phase to the proliferative phase as a result of decreased macrophage recruitment (84). Excessive constant instability and early mechanical loading have been reported to be detrimental to vascular growth (76, 80, 85, 86). Constant less-stable fixation has been reported to prolong the chondral phase, which has been deemed responsible for delayed bridging and increases in the time required for healing in sheep (87, 88) rats (89), and mice (78). On the other hand, superior results have been shown when rigid fixation changed to more flexible fixation at 3 and 4 weeks (73) after surgery compared with at 1 week (90). In addition, there is evidence that loading while matrix deposition and remodelling are ongoing may enhance stabilization through the formation of additional cartilage and bone (79, 86).

**Figure 3:**
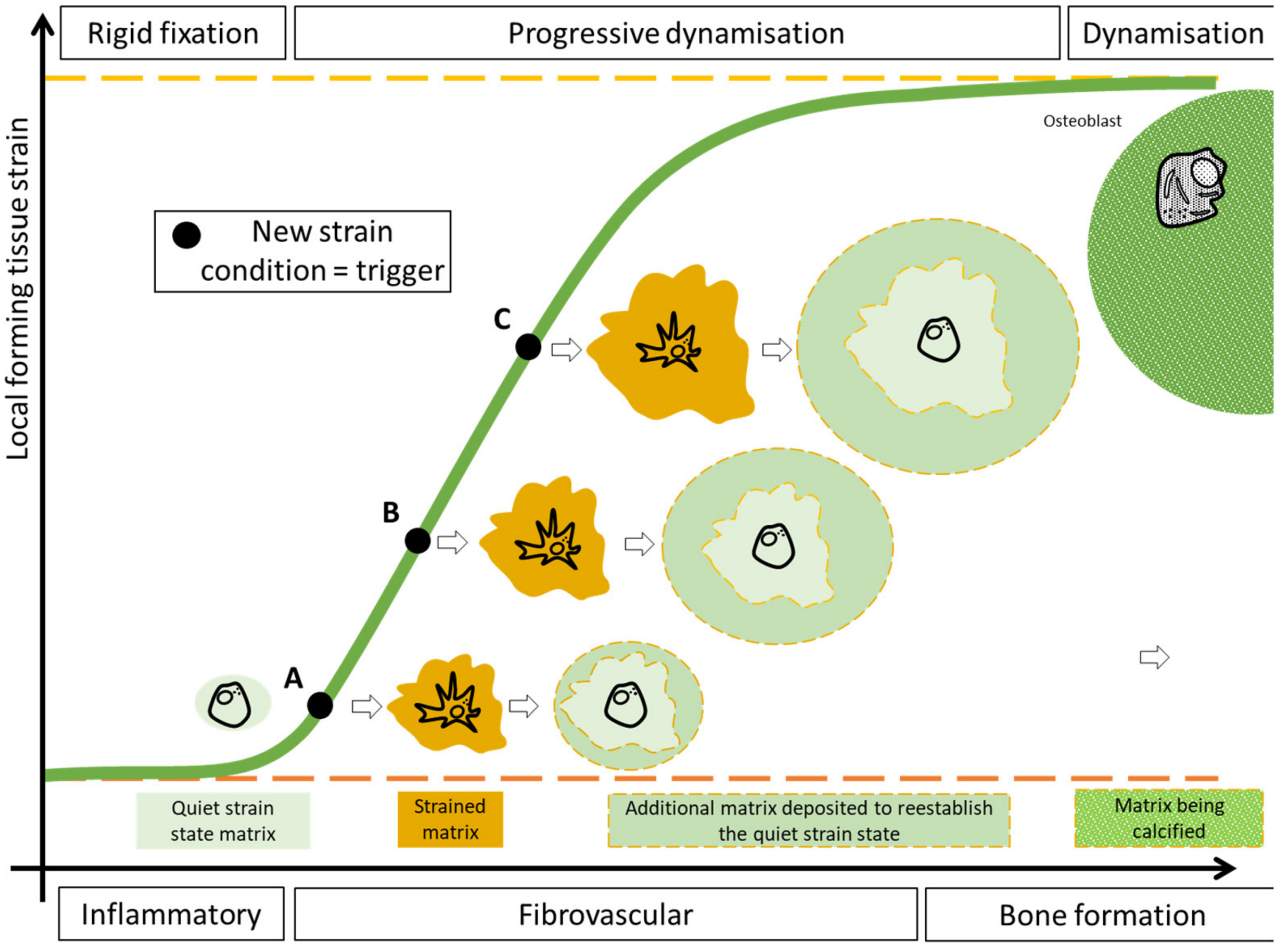
A pictorial representation of the variable fixation concept. On the *x*-axis, simplified, the fracture healing phases in the window of opportunity. On the *y*-axis the average local forming tissue strain. The green sigmoid represent the average tissue strain perceived locally by the cells embedded in the callus. The black dots are samples of average strains taken on a continuous curve. After a period of relatively low deformation a moderate and continuous increase in the local tissue strain is achieved during the fibrovascular phase. A moderate increase in the local tissue strain (**A**) is perceived by the cytoskeleton of the embedded cells and triggers the production of additional extracellular matrix until these same cells return to a quiescent status. The following, moderate, strain increase (**B**,**C**) reactivates the same cells to produce additional extracellular matrix. Such increase in average tissue strain shall be limited in order to allow the deposition of mineral. As the callus matures, the increases in its mechanical properties determines a decrease in the average strain perceived by the embedded cells.

Thus, we believe that such strain-temporal window of opportunity is limited between the inflammatory and the bone formation phases and features an optimal strain level that vary between phases with a level increase between the inflammatory and the callus formation phases (Figure 3). The strain level attained during callus formation should allow for its consolidation and subsequent remodelling.

## 3 How to transition between levels of stability and promote callus formation?

Optimization of fixation flexibility requires an adjustment of strain conditions within a certain range, with a lower limit that ensures initiation of callus production and an upper limit that still allows solid bridging by callus (57). A sudden increase in stimulation aiming at changing the stimulation level between the inflammatory and fibrovascular phase might be suggested. However, an excessive increase in stimulation might constantly damage the tissue in the fracture gap, preventing bridging (91). Progressively transitioning from rigid fixation to a certain degree of dynamization can be used to gradually strain the cells embedded in the forming tissue in the window of opportunity. Such variable fixation has been postulated to foster the callus cells to produce additional extracellular matrix, aiming at re-establishing a neutral strain condition (Figure 3).

**Figure 4:**
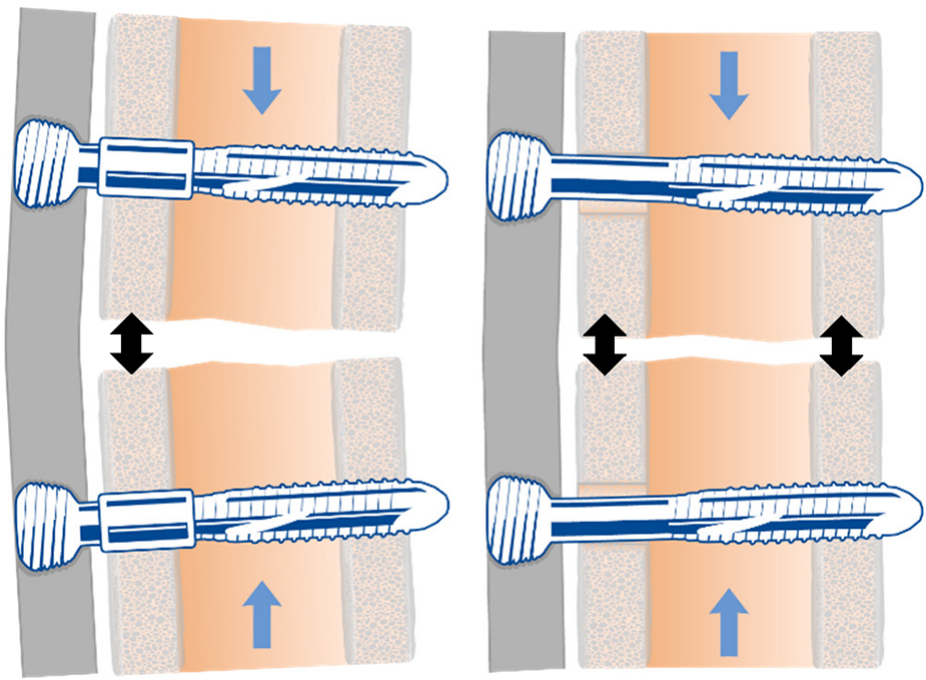
The variable fixation locking screw (VFLS®) at the beginning of the implantation (left) and at the end of sleeve resorption (right). The development of this new implantable device has been driven by the latest advancements in our understanding of the strain-temporal window of opportunity. When the degradation process is completed, the sleeve is entirely absorbed, and the load is entirely shifted from the cis to the trans cortex. The increased working length, determines a decrease in construct stiffness and promotes a more uniform and larger stimulation of the forming bone callus (black arrows).

**Figure 5:**
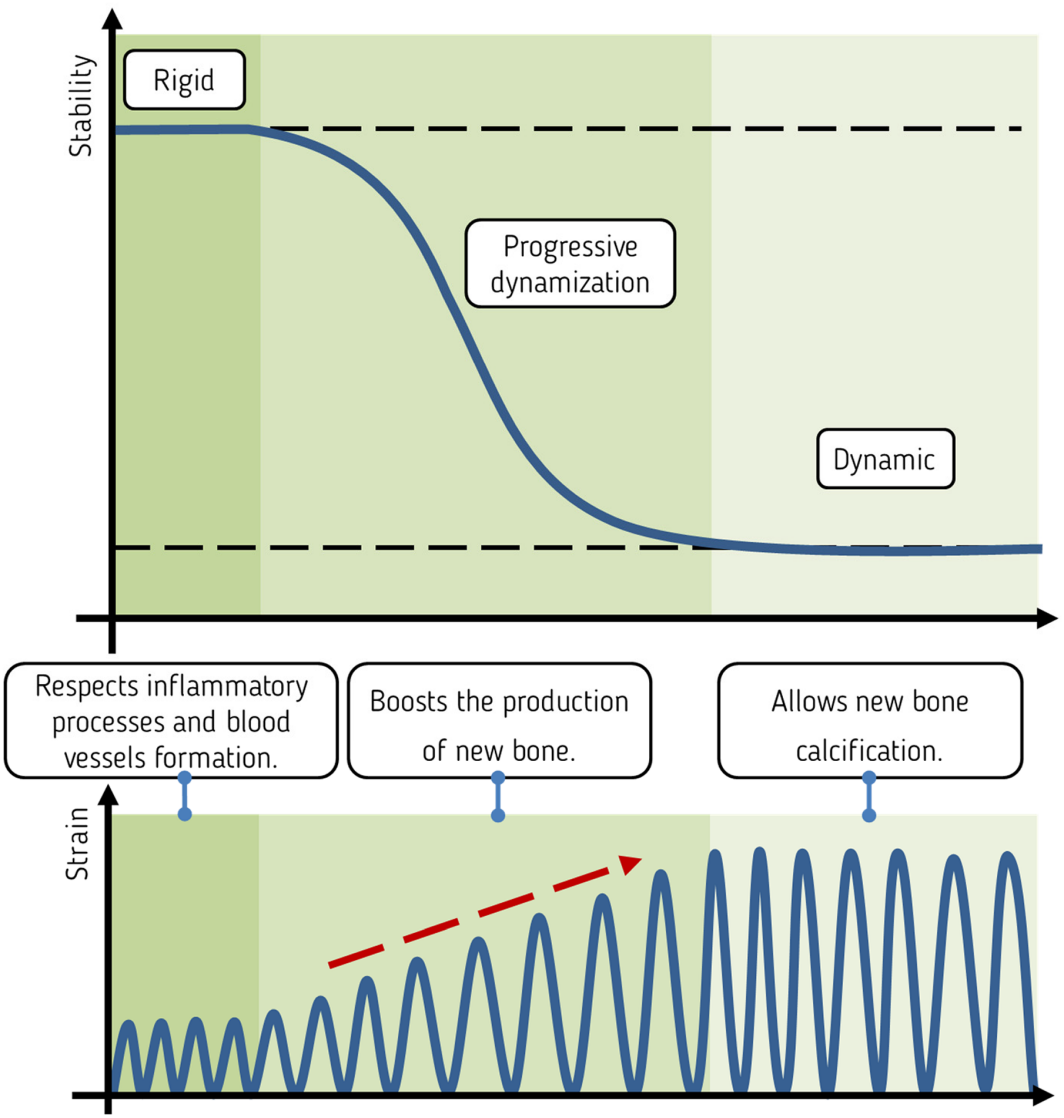
Schematic representation of the effect of a controlled and progressive decrease in construct stability (above) on the average strain perceived by the new forming callus tissue (below) and the respective intent of the design (middle).

To test these hypotheses, a new generation of screws, the Variable Fixation Locking Screws (VFLS®, Biomech Innovations, Nidau, Switzerland), has been developed to stimulate the forming bone tissue in the window of opportunity. The screws incorporate a resorbable sleeve that is positioned to provide support to the cis cortex at implantation, providing stability and motion comparable to a standard locking plate construct with maximum motion at the trans cortex (Figure 4, left). This is followed by a phase in which, due to the degradation of the mechanical properties of the material, the sleeve gradually provides less support to the cis cortex, resulting in a more even distribution of motion between the cis and trans cortices (Figure 4, right). This decrease in stability results in a gradual and limited decrease in construct stiffness, a gradual and limited rise in fracture gap, and thus, callus strain (Figure 5) and a gradual and limited mechanical stimulation of the trans cortex. These screws can replace standard locking screws in cortical bone segments when, according to surgeon decision, boosting fracture healing can be advantageous treating diaphyseal and metaphyseal fractures and osteotomies. When preservation of the local bone supply is necessary, these screws can also be implanted through minimally invasive and percutaneous techniques using standard instrumentation.

**Figure 6:**
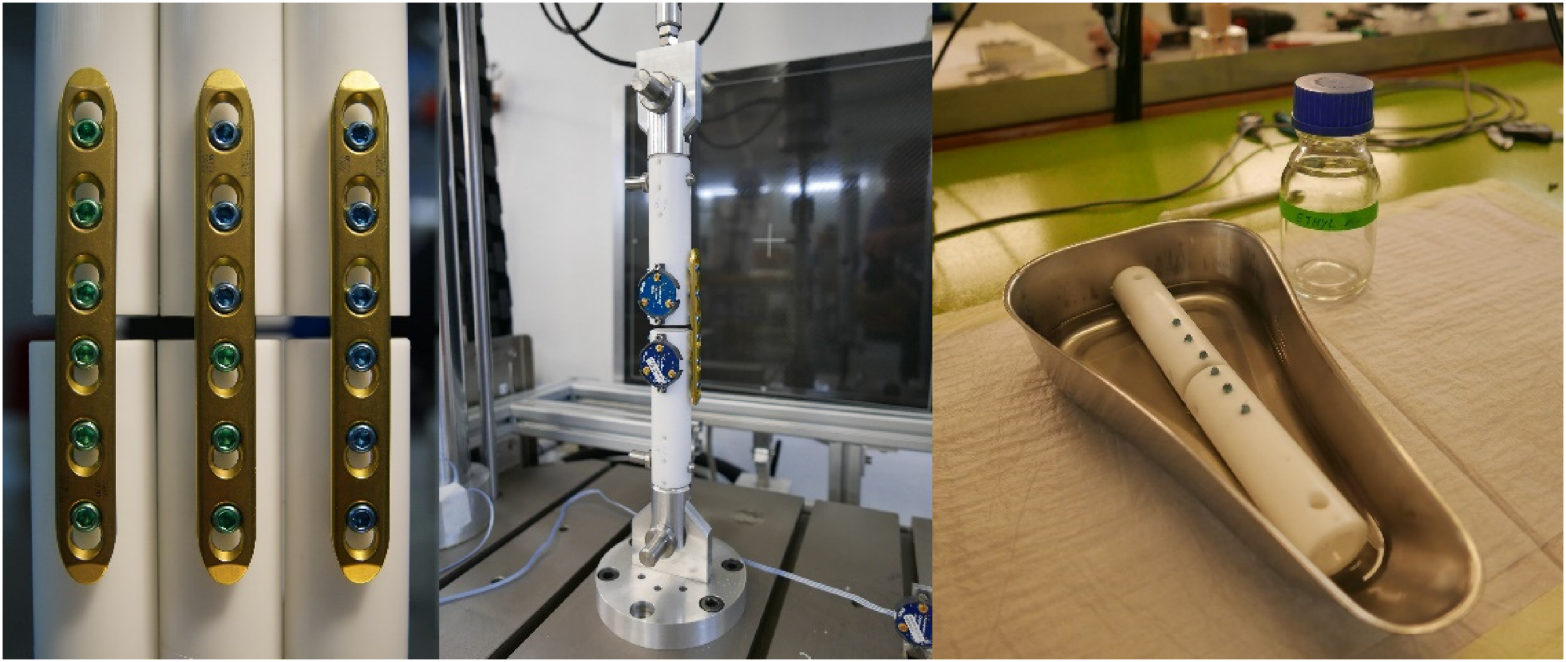
On the left, the three groups tested in a biomechanical investigation: a bone substitute construct featuring three standard locking screws in the proximal and distal segment, a construct featuring three VFLS® in the proximal and three standard locking screws in the distal segment and a construct featuring three VFLS® in the proximal and distal segment. Center: the compression test set up. On the right, the chemical method used to dissolve the resorbable material. Such method allowed testing the same samples with intact and without sleeve without loosening the locking mechanism.

Two studies have been performed using commercially available plates in order to allow direct translation of the results into clinical applications. Initially, a biomechanical study (92) was conducted to assess the interfragmentary stability offered by a combination of a plate and variable fixation locking screws in a simulated fracture-gap model subjected to both compressive and torsional loading. To determine whether the technology was functioning according to its design, the study compared the interfragmentary stability achieved with the intact sleeve to the stability after its chemical dissolution. This was then compared to the stability provided by the same plate with standard locking screws and to a third construct that utilized the same plate in combination with both technologies (Figure 6). The results showed that the VFLS exhibit enhanced tolerance to manual assembly, resulting in more consistent mechanical performance with respect to standard locking screws. The sleeve effectively serves as a centring device for the cis-cortex hole. This critical design feature ensures that all implanted VFLS devices function in parallel, facilitating effective dynamization. When screws are not centred in the cis-cortex hole, they may experience uneven loading, potentially disrupting dynamization by contacting the cis cortex prematurely and increasing the risk of failure. Following implantation, constructs incorporating variable fixation technology, in one or both bone segments, exhibited the same level of rigidity as those achieved with standard locking technology. Moreover, it was demonstrated that these two technologies can be combined without compromising safety. Additionally, as the sleeve undergoes resorption, the stiffness of variable fixation constructs noticeably diminishes, depending on the specific combinations of technologies employed. This reduction in stiffness gradually reaches approximately 15% when mixing standard and variable fixation technologies or 30% when utilizing variable fixation on both sides of the fracture (92). The recorded interfragmentary movements validated that there were comparable displacements in both the cis and trans cortex between standard and Variable Fixation constructs when the sleeve was intact. However, the resorption of the sleeve dynamized the gap leading to a substantial increase in displacement at the trans and, even more, at the cis cortex (92). Mixing Variable Fixation with standard locking technology the increase ranged between 12% and 20% at the trans-cortex and between 50% and 60% at the cis-cortex. Using Variable fixation on both segments, sleeve degradation resulted in an increase of around 20%–37% in trans-cortex and approximately 70%–125% in cis-cortex axial displacements.

**Figure 7:**
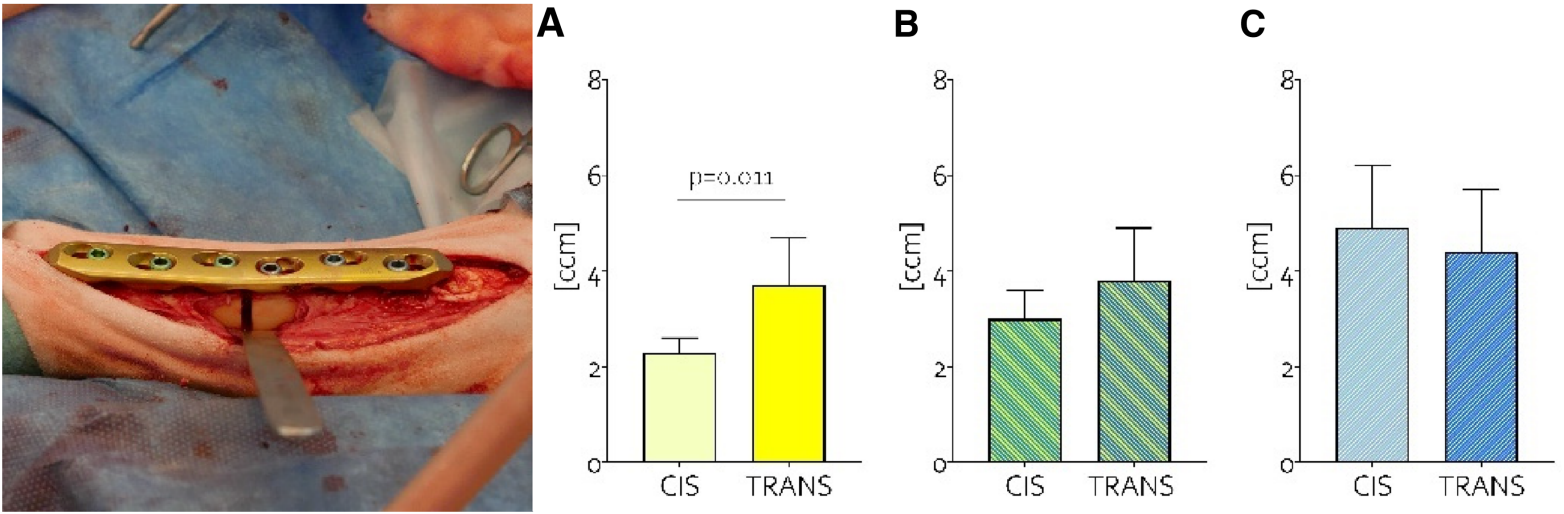
On the left, the 3 mm osteotomy fixed with a locking plate and, in this case, variable fixation on the proximal and standard locking screws on the distal segment. On the right the amount of callus volume (ccm) detected at the cis and trans cortices in the groups featuring standard locking technology on both bone segments (**A**), the group with mixed technologies (**B**) and the group featuring Variable Fixation on both bone segments (**C**) These data provide evidence that the dynamization tool was effective in negating the significant cis-trans difference detected when using standard locking technology. Moreover, when the variable fixation stimulation was doubled, it resulted in a notable increase in cis callus volume without causing an excessive rise in trans callus volume.

The second study (93) was a preclinical investigation whose results provided strong validation for the thesis established in the biomechanical study (92) and the concept of Variable Fixation. There, the formation of callus was investigated along the entire bone and at the fracture site in three groups of sheep. These animals had 3 mm tibial osteotomies that were stabilized using configurations similar to those assessed in the biomechanical investigation, namely standard locking screws on both bone segments, Variable Fixation Locking Screws on one bone segment and standard locking screws on the other and Variable Fixation Locking Screws on both bone segments (Figure 7). The results demonstrated that Variable fixation promoted the formation of a substantially larger amount of bone callus compared to standard rigid fixation. The gradual reduction in stiffness and a targeted change in interfragmentary displacements influenced the generation and spatial distribution of bone callus. In the whole bone this was demonstrated by a 40% larger callus with similar mineral density in the group featuring variable fixation in one bone segment. The responsiveness of mechanobiological signals was reaffirmed at the fracture site, where, in comparison to standard locking technology, variable fixation resulted in a +30% larger callus on the cis side and a more evenly distributed callus between the cortices (93). Remarkably, the findings from the same study demonstrated that the extent of variable fixation applied to the fracture gap has a biological effect. Using variable fixation on both bone segments doubled the magnitude of progressive dynamization and promoted the formation of an even larger bone callus. Nonetheless, in case of a 3 mm gap, this came with a slight decrease (about 10%) in mineral density. This confirms the presence of a strain dimension within the “window of opportunity”. Like all implantable devices, the usage of variable fixation should always be adjusted to the mass of the patient, the stiffness of the chosen bone plate and size of the fracture gap (93).

## 4 The bone is an organ

Bones possess a remarkable capacity for self-repair that extends throughout their entire structure of the organ. In the event of a fracture, it is common for the bone to preserve adjacent, healthy segments that encompass all the essential cellular elements, signalling pathways, and tissues required to actively initiate and proficiently conclude the process of fracture healing (94). Variable Fixation has been designed with the intention of mechanically stimulating these undamaged bone segments, thereby promoting the activation of these healthy portions to participate in the formation of bone callus and contribute to the comprehensive restoration of the fractured bone (93).

**Figure 8:**
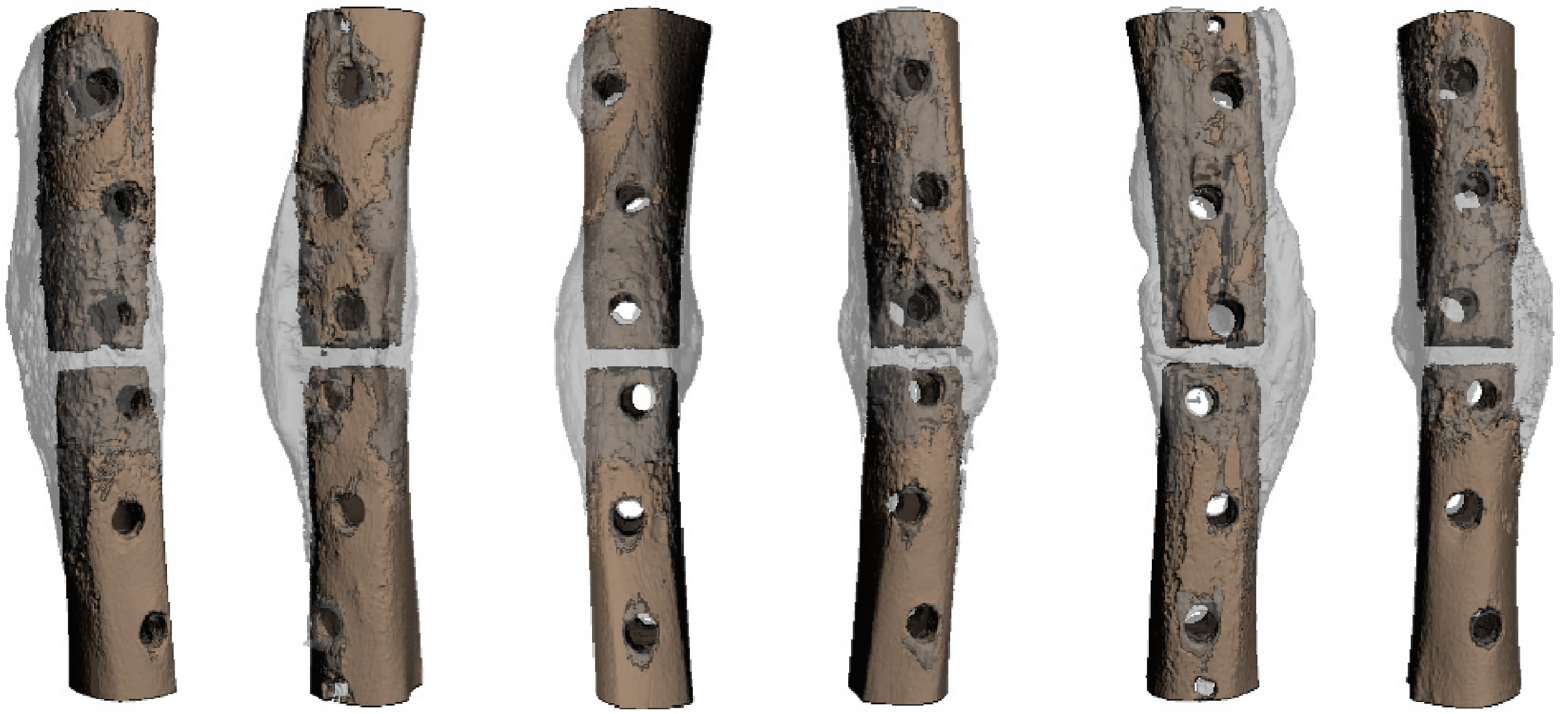
3d reconstruction of the 3 mm tibia osteotomies stabilized using a locking plate, with variable fixation screws applied to the proximal segment and standard locking screws on the distal segment. In this visual representation, the native cortical bone is depicted in brown, while the callus is shown in grey. It's worth noting the increased volume of callus formation in all proximal bone segments where variable fixation locking screws were used, indicating a response from the periosteum to altered loading conditions (93).

Although studies have suggested a potential systemic recruitment of skeletal stem cells for bone repair (95), Duchamp et al. have shown with a renal capsule model that the systemic recruitment of cells does not occur for endogenous bone repair and that the cells forming the fracture callus are all recruited locally (96). During endochondral ossification, skeletal stem cells within the endosteum and periosteum segregate into distinct bone compartments, each adopting distinct functions within adult bones (96). Within the bone marrow compartment, bone marrow stem cells serve as the niche for hematopoietic stem cells, overseeing bone remodelling and playing roles in immunomodulation and paracrine functions in the context of bone maintenance and repair (96). Within the periosteum compartment, periosteal cells play a more direct role in bone repair, participating in the formation of cartilage and bone within the callus (96). Bone marrow stem cells are now occasionally used in orthopaedics. However, in mice, the periosteal skeletal stem cells have shown higher bone regenerative potential compared to bone marrow stem cells (96). When responding to injury, the activation of skeletal stem cells is orchestrated through the upregulation of various extracellular matrix proteins. Periostin, among these proteins, plays a crucial role in facilitating proper bone repair by regulating both cell-cell and cell-matrix interactions. It exhibits high expression in periosteal cells during development and is notably prevalent in adult tissues subjected to mechanical stress (97). This has been indirectly confirmed in the preclinical study in sheep (93), where the progressive stimulation generated by Variable Fixation screws on the trans cortex led to the activation of both tissues, resulting in the reinforcement of the fracture site and the generation of additional periosteal bone callus (Figure 8). It is intriguing to note that this effect was observable at a distance from the fracture gap, underscoring the remarkable engraftment capacity of periosteal cells when transplanted at an injury site, particularly their high capability to form cartilage and bone during the process of skeletal regeneration (96).

## 5 Conclusions and future directions

We propose the concept of a “strain-temporal window of opportunity” as a conceptual approach to the design of implants that potentially promote bone formation. Emerging insights from mechanobiology suggest that there are specific time and strain parameters that create a window of opportunity during which the process of fracture healing can effectively unfold. Beyond this window, fracture healing may be incomplete or significantly disrupted. The commencement of this temporal window appears to be individually tailored based on the injury and the patient. Optimal local tissue strain conditions vary across different phases, with an increase between the inflammatory and fibrovascular phases. Finally, recent advancements in our understanding of skeletal stem cells highlight the pivotal role of the periosteum in offering cells with the highest bone regenerative potential. Constructs composed of medical devices that gradually strain the developing tissue while simultaneously stimulating the periosteum seem to hold the potential to enhance bone callus formation and ameliorate the adverse effects of healing complications. Clearly, patients related factors, like metabolic disease, have a systemic effect and may cause delayed or non union even when the stimulation of the forming tissue falls in the postulated window of opportunity.

The Variable Fixation Locking Screw (VFLS®) may represent a step forward in angular stable plate osteosynthesis. It upholds the inherent benefits of angular stability while incorporating a safe and consistent dynamization approach, informed by the latest insights from mechanobiology. Acting on the screw, the surgeon has the opportunity to finely adjust the rigidity of osteosynthesis procedures in accordance with established fracture treatment principles.

Today, we are actively engaged in the meticulous monitoring of the healing progress in specific clinical cases involving fractures and osteotomies. Our goal is to gain a deeper understanding of whether this approach holds the potential to enhance the success rate of osteosynthesis procedures. Through this ongoing research, we aim to refine and optimize fracture treatment techniques, ultimately resulting in improved patient outcomes and potential cost savings in healthcare.

## References

[1] Steppe L Megafu M Tschaffon-Müller MEA Ignatius A Haffner-Luntzer M. Fracture healing research: recent insights. Bone Rep. (2023) 19:101686. 10.1016/j.bonr.2023.101686 38163010 PMC10757288

[2] Wu A-M Bisignano C James SL Abady GG Abedi A Abu-Gharbieh E Global, regional, and national burden of bone fractures in 204 countries and territories, 1990–2019: a systematic analysis from the global burden of disease study 2019. Lancet Healthy Longev. (2021) 2(9):e580–92. 10.1016/S2666-7568(21)00172-0 34723233 PMC8547262

[3] Bonafede M Espindle D Bower AG. The direct and indirect costs of long bone fractures in a working age us population. J Med Econ. (2013) 16(1):169–78. 10.3111/13696998.2012.737391 23035626

[4] Court-Brown CM Clement N. Four score years and ten: an analysis of the epidemiology of fractures in the very elderly. Injury. (2009) 40(10):1111–4. 10.1016/j.injury.2009.06.011 19596316

[5] Court-Brown CM Caesar B. Epidemiology of adult fractures: a review. Injury. (2006) 37(8):691–7. 10.1016/j.injury.2006.04.130 16814787

[6] Dahabreh Z Calori GM Kanakaris NK Nikolaou VS Giannoudis PV. A cost analysis of treatment of tibial fracture nonunion by bone grafting or bone morphogenetic protein-7. Int Orthop. (2009) 33(5):1407–14. 10.1007/s00264-008-0709-6 19052743 PMC2899110

[7] Bell A Templeman D Weinlein JC. Nonunion of the femur and tibia: an update. Orthop Clin North Am. (2016) 47(2):365–75. 10.1016/j.ocl.2015.09.010 26772945

[8] Antonova E Le TK Burge R Mershon J. Tibia shaft fractures: costly burden of nonunions. BMC Musculoskelet Disord. (2013) 14:42. 10.1186/1471-2474-14-42 23351958 PMC3573940

[9] Hemmann P Friederich M Körner D Klopfer T Bahrs C. Changing epidemiology of lower extremity fractures in adults over a 15-year period—a national hospital discharge registry study. BMC Musculoskelet Disord. (2021) 22(1):456. 10.1186/s12891-021-04291-9 34011331 PMC8135150

[10] Pagani NR Varady NH Chen AF Rajaee SS Kavolus JJ. Nationwide analysis of lower extremity periprosthetic fractures. J Arthroplasty. (2021) 36(1):317–24. 10.1016/j.arth.2020.07.050 32826143

[11] Cohen S. Statistical brief #359: the concentration of health care expenditures and related expenses for costly medical conditions (2009).9. 29281226

[12] Einhorn TA Gerstenfeld LC. Fracture healing: mechanisms and interventions. Nat Rev Rheumatol. (2015) 11(1):45–54. 10.1038/nrrheum.2014.164 25266456 PMC4464690

[13] Gómez-Barrena E Rosset P Lozano D Stanovici J Ermthaller C Gerbhard F. Bone fracture healing: cell therapy in delayed unions and nonunions. Bone. (2015) 70:93–101. 10.1016/j.bone.2014.07.033 25093266

[14] Calori GM Mazza EL Mazzola S Colombo A Giardina F Romanò F Non-unions. Clin Cases Miner Bone Metab. (2017) 14(2):186–8. 10.11138/ccmbm/2017.14.1.186 29263731 PMC5726207

[15] Hellwinkel JE Miclau T Provencher MT Bahney CS Working ZM. The life of a fracture: biologic progression, healing gone awry, and evaluation of union. JBJS Rev. (2020) 8(8):e1900221. 10.2106/JBJS.RVW.19.00221 32796195 PMC11147169

[16] Calori GM Mazza E Colombo M Ripamonti C Tagliabue L. Treatment of long bone non-unions with polytherapy: indications and clinical results. Injury. (2011) 42(6):587–90. 10.1016/j.injury.2011.03.046 21524745

[17] Starčević N Karačić A. Infected nonunion of the distal femur in the elderly with bone loss: case report and treatment options. Case Rep Orthop. (2021) 2021:3530297. 10.1155/2021/3530297 34580614 PMC8464431

[18] Walter N Kerschbaum M Pfeifer C Popp D Freigang V Hinterberger T Long-term patient-related quality of life after successfully treated aseptic non-unions of the long bones. Injury. (2021) 52(7):1880–5. 10.1016/j.injury.2021.04.041 33910685

[19] Hak DJ Fitzpatrick D Bishop JA Marsh JL Tilp S Schnettler R Delayed union and nonunions: epidemiology, clinical issues, and financial aspects. Injury. (2014) 45(Suppl 2):S3–7. 10.1016/j.injury.2014.04.002 24857025

[20] Copuroglu C Calori GM Giannoudis PV. Fracture non-union: who is at risk? Injury. (2013) 44(11):1379–82. 10.1016/j.injury.2013.08.003 24035757

[21] Calori GM Colombo M Mazza EL Mazzola S Malagoli E Marelli N Validation of the non-union scoring system in 300 long bone non-unions. Injury. (2014) 45(Suppl 6):S93–7. 10.1016/j.injury.2014.10.030 25457326

[22] Ernst M Richards RG Windolf M. Smart implants in fracture care—only buzzword or real opportunity? Injury. (2021) 52(Suppl 2):S101–5. 10.1016/j.injury.2020.09.026 32980139

[23] Windolf M Heumann M Varjas V Constant C Ernst M Richards RG Continuous rod load monitoring to assess spinal fusion status-pilot *in vivo* data in sheep. Medicina. (2022) 58(7):899. 10.3390/medicina58070899 PMC9319051 35888618

[24] Windolf M Varjas V Gehweiler D Schwyn R Arens D Constant C Continuous implant load monitoring to assess bone healing Status-evidence from animal testing. Medicina. (2022) 58(7):858. 10.3390/medicina58070858 PMC9321316 35888576

[25] Ghiasi MS Chen J Vaziri A Rodriguez EK Nazarian A. Bone fracture healing in mechanobiological modeling: a review of principles and methods. Bone Rep. (2017) 6:87–100. 10.1016/j.bonr.2017.03.002 28377988 PMC5365304

[26] Glatt V Evans CH Tetsworth K. A concert between biology and biomechanics: the influence of the mechanical environment on bone healing. Front Physiol. (2016) 7:678. 10.3389/fphys.2016.00678 28174539 PMC5258734

[27] Thompson Z Miclau T Hu D Helms JA. A model for intramembranous ossification during fracture healing. J Orthop Res. (2002) 20(5):1091–8. 10.1016/S0736-0266(02)00017-7 12382977

[28] Augat P Hollensteiner M Rüden C. The role of mechanical stimulation in the enhancement of bone healing. Injury. (2021) 52(Suppl 2):S78–83. 10.1016/j.injury.2020.10.009 33041020

[29] Wittkowske C Reilly GC Lacroix D Perrault CM. *In vitro* bone cell models: impact of fluid shear stress on bone formation. Front Bioeng Biotechnol. (2016) 4:87. 10.3389/fbioe.2016.00087 27896266 PMC5108781

[30] Witt F Duda GN Bergmann C Petersen A. Cyclic mechanical loading enables solute transport and oxygen supply in bone healing: an *in vitro* investigation. Tissue Eng Part A. (2014) 20(3-4):486–93. 10.1089/ten.TEA.2012.0678 24125527

[31] Bottlang M Feist F. Biomechanics of far cortical locking. J Orthop Trauma. (2011) 25(Suppl 1):S21–8. 10.1097/BOT.0b013e318207885b 21248556 PMC3062510

[32] Jaeblon T. Biomechanics of far cortical locking. J Orthop Trauma. (2011) 25(6):e60. 10.1097/01.bot.0000398506.96521.7f 21577061

[33] Bottlang M Doornink J Fitzpatrick DC Madey SM. Far cortical locking can reduce stiffness of locked plating constructs while retaining construct strength. J Bone Joint Surg Am. (2009) 91(8):1985–94. 10.2106/JBJS.H.01038 19651958 PMC2714811

[34] Bottlang M Lesser M Koerber J Doornink J von Rechenberg B Augat P Far cortical locking can improve healing of fractures stabilized with locking plates. J Bone Joint Surg Am. (2010) 92(7):1652–60. 10.2106/JBJS.I.01111 20595573 PMC2897208

[35] Dobele S Horn C Eichhorn S Buchholtz A Lenich A Burgkart R The dynamic locking screw (Dls) can increase interfragmentary motion on the near cortex of locked plating constructs by reducing the axial stiffness. Langenbecks Arch Surg. (2010) 395(4):421–8. 10.1007/s00423-010-0636-z 20358382

[36] Freude T Schroter S Gonser CE Stockle U Acklin YP Hontzsch D Controlled dynamic stability as the next step in “biologic plate osteosynthesis”—a pilot prospective observational cohort study in 34 patients with distal tibia fractures. Patient Saf Surg. (2014) 8(1):3. 10.1186/1754-9493-8-3 24447901 PMC3939631

[37] Freude T Schroter S Kraus TM Hontzsch D Stockle U Dobele S. Dynamic locking screw 5.0–first clinical experience. Z Orthop Unfall. (2013) 151(3):284–90. 10.1055/s-0032-1328521 23771332

[38] Plecko M Lagerpusch N Andermatt D Frigg R Koch R Sidler M The dynamisation of locking plate osteosynthesis by means of dynamic locking screws (Dls)-an experimental study in sheep. Injury. (2013) 44(10):1346–57. 10.1016/j.injury.2012.10.022 23182750

[39] Richter H Plecko M Andermatt D Frigg R Kronen PW Klein K Dynamization at the near cortex in locking plate osteosynthesis by means of dynamic locking screws: an experimental study of transverse tibial osteotomies in sheep. J Bone Joint Surg Am. (2015) 97(3):208–15. 10.2106/JBJS.M.00529 25653321

[40] Bottlang M Tsai S Bliven EK von Rechenberg B Klein K Augat P Dynamic stabilization with active locking plates delivers faster, stronger, and more symmetric fracture-healing. J Bone Joint Surg Am. (2016) 98(6):466–74. 10.2106/JBJS.O.00705 26984914 PMC4788849

[41] Tsai S Fitzpatrick DC Madey SM Bottlang M. Dynamic locking plates provide symmetric axial dynamization to stimulate fracture healing. J Orthop Res. (2015) 33(8):1218–25. 10.1002/jor.22881 25721801

[42] Epari DR Gurung R Hofmann-Fliri L Schwyn R Schuetz M Windolf M. Biphasic plating improves the mechanical performance of locked plating for distal femur fractures. J Biomech. (2021) 115:110192. 10.1016/j.jbiomech.2020.110192 33385868

[43] Hofmann-Fliri L Epari DR Schwyn R Zeiter S Windolf M. Biphasic plating—*in vivo* study of a novel fixation concept to enhance mechanobiological fracture healing. Injury. (2020) 51(8):1751–8. 10.1016/j.injury.2020.04.032 32536529

[44] Linn MS McAndrew CM Prusaczyk B Brimmo O Ricci WM Gardner MJ. Dynamic locked plating of distal femur fractures. J Orthop Trauma. (2015) 29(10):447–50. 10.1097/BOT.0000000000000315 25714439

[45] Wähnert D Greiner J Brianza S Kaltschmidt C Vordemvenne T Kaltschmidt B. Strategies to improve bone healing: innovative surgical implants meet nano-/micro-topography of bone scaffolds. Biomedicines. (2021) 9(7):21. 10.3390/biomedicines9070746 PMC8301359 34203437

[46] Gardner ROE Bates JH Ng'oma E Harrison WJ. Fracture union following internal fixation in the hiv population. Injury. (2013) 44(6):830–3. 10.1016/j.injury.2012.11.024 23267724

[47] Gortler H Rusyn J Godbout C Chahal J Schemitsch EH Nauth A. Diabetes and healing outcomes in lower extremity fractures: a systematic review. Injury. (2018) 49(2):177–83. 10.1016/j.injury.2017.11.006 29162268

[48] Mills L Tsang J Hopper G Keenan G Simpson AHRW. The multifactorial aetiology of fracture nonunion and the importance of searching for latent infection. Bone Joint Res. (2016) 5(10):512–9. 10.1302/2046-3758.510.BJR-2016-0138 27784669 PMC5108351

[49] Reahl GB Gerstenfeld L Kain M. Epidemiology, clinical assessments, and current treatments of nonunions. Curr Osteoporos Rep. (2020) 18(3):157–68. 10.1007/s11914-020-00575-6 32318988

[50] Santolini E West R Giannoudis PV. Risk factors for long bone fracture non-union: a stratification approach based on the level of the existing scientific evidence. Injury. (2015) 46(Suppl):S8–19. 10.1016/S0020-1383(15)30049-8 26747924

[51] Zura R Xiong Z Einhorn T Watson JT Ostrum RF Prayson MJ Epidemiology of fracture nonunion in 18 human bones. JAMA Surg. (2016) 151(11):e162775. 10.1001/jamasurg.2016.2775 27603155

[52] Hurtgen BJ Ward CL Garg K Pollot BE Goldman SM McKinley TO Severe muscle trauma triggers heightened and prolonged local musculoskeletal inflammation and impairs adjacent tibia fracture healing. J Musculoskelet Neuronal Interact. (2016) 16(2):122–34. ; . 27282456 PMC5114355

[53] O'Halloran K Coale M Costales T Zerhusen T Castillo RC Nascone JW Will my tibial fracture heal? Predicting nonunion at the time of definitive fixation based on commonly available variables. Clin Orthop Relat Res. (2016) 474(6):1385–95. 10.1007/s11999-016-4821-4 27125823 PMC4868164

[54] Schmal H Brix M Bue M Ekman A Ferreira N Gottlieb H Nonunion—consensus from the 4th annual meeting of the Danish orthopaedic trauma society. EFORT Open Rev. (2020) 5(1):46–57. 10.1302/2058-5241.5.190037 32071773 PMC7017598

[55] Pearson RG Clement RGE Edwards KL Scammell BE. Do smokers have greater risk of delayed and non-union after fracture, osteotomy and arthrodesis? A systematic review with meta-analysis. BMJ Open. (2016) 6(11):e010303. 10.1136/bmjopen-2015-010303 28186922 PMC5129177

[56] Richards CJ Graf KW Mashru RP. The effect of opioids, alcohol, and nonsteroidal anti-inflammatory drugs on fracture union. Orthop Clin North Am. (2017) 48(4):433–43. 10.1016/j.ocl.2017.06.002 28870304

[57] Hente RW Perren SM. Tissue deformation controlling fracture healing. J Biomech. (2021) 125:110576. 10.1016/j.jbiomech.2021.110576 34171609

[58] Augat P Simpson H. Enhancement of fracture healing. Injury. (2021) 52(Suppl 2):S1–2. 10.1016/j.injury.2021.05.023 34099104

[59] Claes L. Improvement of clinical fracture healing—what can be learned from mechano-biological research? J Biomech. (2021) 115:110148. 10.1016/j.jbiomech.2020.110148 33341439

[60] Menger MM Laschke MW Nussler AK Menger MD Histing T. The vascularization paradox of non-union formation. Angiogenesis. (2022) 25(3):279–90. 10.1007/s10456-022-09832-x 35165821 PMC9249698

[61] Patel ZS Young S Tabata Y Jansen JA Wong MEK Mikos AG. Dual delivery of an angiogenic and an osteogenic growth factor for bone regeneration in a critical size defect model. Bone. (2008) 43(5):931–40. 10.1016/j.bone.2008.06.019 18675385 PMC3014108

[62] Kaipel M Schützenberger S Schultz A Ferguson J Slezak P Morton TJ Bmp-2 but not Vegf or Pdgf in fibrin matrix supports bone healing in a delayed-union rat model. J Orthop Res. (2012) 30(10):1563–9. 10.1002/jor.22132 22508566

[63] Kempen DHR Lu L Heijink A Hefferan TE Creemers LB Maran A Effect of local sequential Vegf and Bmp-2 delivery on ectopic and orthotopic bone regeneration. Biomaterials. (2009) 30(14):2816–25. 10.1016/j.biomaterials.2009.01.031 19232714

[64] Geuze RE Theyse LFH Kempen DHR Hazewinkel HAW Kraak HYA Oner FC A differential effect of bone morphogenetic protein-2 and vascular endothelial growth factor release timing on osteogenesis at ectopic and orthotopic sites in a large-animal model. Tissue Eng, Part A. (2012) 18(19-20):2052–62. 10.1089/ten.TEA.2011.0560 22563713 PMC3463278

[65] Hernández A Reyes R Sánchez E Rodríguez-Évora M Delgado A Evora C. *In vivo* osteogenic response to different ratios of Bmp-2 and Vegf released from a biodegradable porous system. J Biomed Mater Res A. (2012) 100(9):2382–91. 10.1002/jbm.a.34183 22528545

[66] Orth M Altmeyer MAB Scheuer C Braun BJ Holstein JH Eglin D Effects of locally applied adipose tissue-derived microvascular fragments by thermoresponsive hydrogel on bone healing. Acta Biomater. (2018) 77:201–11. 10.1016/j.actbio.2018.07.029 30030175

[67] Chung Y-G Bishop AT Giessler GA Suzuki O Platt JL Pelzer M Surgical angiogenesis: a new approach to maintain osseous viability in xenotransplantation. Xenotransplantation. (2010) 17(1):38–47. 10.1111/j.1399-3089.2009.00563.x 20149187

[68] Allsopp BJ Hunter-Smith DJ Rozen WM. Vascularized versus nonvascularized bone grafts: what is the evidence? Clin Orthop Relat Res. (2016) 474(5):1319–27. 10.1007/s11999-016-4769-4 26932740 PMC4814434

[69] Hirche C Xiong L Heffinger C Münzberg M Fischer S Kneser U Vascularized versus non-vascularized bone grafts in the treatment of scaphoid non-union. J Orthop Surg. (2017) 25(1):2309499016684291. 10.1177/2309499016684291 28125938

[70] Perren SM. Evolution of the internal fixation of long bone fractures. The scientific basis of biological internal fixation: choosing a new balance between stability and biology. J Bone Joint Surg Br. (2002) 84(8):1093–110. 10.1302/0301-620X.84B8.0841093 12463652

[71] Pape H-C Moore EE McKinley T Sauaia A. Pathophysiology in patients with polytrauma. Injury. (2022) 53(7):2400–12. 10.1016/j.injury.2022.04.009 35577600

[72] Pfeifer R Kalbas Y Coimbra R Leenen L Komadina R Hildebrand F Indications and interventions of damage control orthopedic surgeries: an expert opinion survey. Eur J Trauma Emerg Surg. (2021) 47(6):2081–92. 10.1007/s00068-020-01386-1 32458046

[73] Claes L Blakytny R Besse J Bausewein C Ignatius A Willie B. Late dynamization by reduced fixation stiffness enhances fracture healing in a rat femoral osteotomy model. J Orthop Trauma. (2011) 25(3):169–74. 10.1097/BOT.0b013e3181e3d994 21321508

[74] Willie BM Blakytny R Glöckelmann M Ignatius A Claes L. Temporal variation in fixation stiffness affects healing by differential cartilage formation in a rat osteotomy model. Clin Orthop Relat Res. (2011) 469(11):3094–101. 10.1007/s11999-011-1866-2 21416204 PMC3183193

[75] Henderson CE Kuhl LL Fitzpatrick DC Marsh JL. Locking plates for distal femur fractures: is there a problem with fracture healing? J Orthop Trauma. (2011) 25(Suppl 1):S8–14. 10.1097/BOT.0b013e3182070127 21248560

[76] Klein P Schell H Streitparth F Heller M Kassi JP Kandziora F The initial phase of fracture healing is specifically sensitive to mechanical conditions. J Orthop Res. (2003) 21(4):662–9. 10.1016/S0736-0266(02)00259-0 12798066

[77] Epari DR Taylor WR Heller MO Duda GN. Mechanical conditions in the initial phase of bone healing. Clin Biomech. (2006) 21(6):646–55. 10.1016/j.clinbiomech.2006.01.003 16513229

[78] Gardner MJ van der Meulen MCH Demetrakopoulos D Wright TM Myers ER Bostrom MP. *In vivo* cyclic axial compression affects bone healing in the mouse tibia. J Orthop Res. (2006) 24(8):1679–86. 10.1002/jor.20230 16788988 PMC2944415

[79] Liu C Carrera R Flamini V Kenny L Cabahug-Zuckerman P George BM Effects of mechanical loading on cortical defect repair using a novel mechanobiological model of bone healing. Bone. (2018) 108:145–55. 10.1016/j.bone.2017.12.027 29305998 PMC8262576

[80] Claes L Eckert-Hübner K Augat P. The effect of mechanical stability on local vascularization and tissue differentiation in callus healing. J Orthop Res. (2002) 20(5):1099–105. 10.1016/S0736-0266(02)00044-X 12382978

[81] Wallace AL Draper ER Strachan RK McCarthy ID Hughes SP. The vascular response to fracture micromovement. Clin Orthop Relat Res. (1994) 301:281–90. 10.1097/00003086-199404000-00044 8156689

[82] Le AX Miclau T Hu D Helms JA. Molecular aspects of healing in stabilized and non-stabilized fractures. J Orthop Res. (2001) 19(1):78–84. 10.1016/S0736-0266(00)00006-1 11332624

[83] Miclau T Lu C Thompson Z Choi P Puttlitz C Marcucio R Effects of delayed stabilization on fracture healing. J Orthop Res. (2007) 25(12):1552–8. 10.1002/jor.20435 17593540 PMC2844641

[84] Hankemeier S Grässel S Plenz G Spiegel HU Bruckner P Probst A. Alteration of fracture stability influences chondrogenesis, osteogenesis and immigration of macrophages. J Orthop Res. (2001) 19(4):531–8. 10.1016/S0736-0266(00)00044-9 11518257

[85] Lienau J Schell H Duda GN Seebeck P Muchow S Bail HJ. Initial vascularization and tissue differentiation are influenced by fixation stability. J Orthop Res. (2005) 23(3):639–45. 10.1016/j.orthres.2004.09.006 15885486

[86] Boerckel JD Uhrig BA Willett NJ Huebsch N Guldberg RE. Mechanical regulation of vascular growth and tissue regeneration *in vivo*. Proc Natl Acad Sci U S A. (2011) 108(37):E674–80. 10.1073/pnas.1107019108 21876139 PMC3174614

[87] Epari DR Schell H Bail HJ Duda GN. Instability prolongs the chondral phase during bone healing in sheep. Bone. (2006) 38(6):864–70. 10.1016/j.bone.2005.10.023 16359937

[88] Schell H Epari DR Kassi JP Bragulla H Bail HJ Duda GN. The course of bone healing is influenced by the initial shear fixation stability. J Orthop Res. (2005) 23(5):1022–8. 10.1016/j.orthres.2005.03.005 15878254

[89] Wehner T Gruchenberg K Bindl R Recknagel S Steiner M Ignatius A Temporal delimitation of the healing phases via monitoring of fracture callus stiffness in rats. J Orthop Res. (2014) 32(12):1589–95. 10.1002/jor.22721 25183200

[90] Claes L Blakytny R Göckelmann M Schoen M Ignatius A Willie B. Early dynamization by reduced fixation stiffness does not improve fracture healing in a rat femoral osteotomy model. J Orthop Res. (2009) 27(1):22–7. 10.1002/jor.20712 18634011

[91] Hente R Füchtmeier B Schlegel U Ernstberger A Perren SM. The influence of cyclic compression and distraction on the healing of experimental tibial fractures. J Orthop Res. (2004) 22(4):709–15. 10.1016/j.orthres.2003.11.007 15183425

[92] Frank A Brianza S Plecko M Raschke MJ Wähnert D. Variable fixation technology provides rigid as well as progressive dynamic fixation: a biomechanical investigation. J Bone Joint Surg Am. (2020) 102(20):e115. 10.2106/JBJS.19.01302 33086351

[93] Plecko M Klein K Planzer K Wähnert D Behm P Ferguson SJ Variable fixation promotes callus formation: an experimental study on transverse tibial osteotomies stabilized with locking plates. BMC Musculoskelet Disord. (2020) 21(1):806. 10.1186/s12891-020-03781-6 33272239 PMC7713143

[94] Moore ER Maridas DE Gamer L Chen G Burton K Rosen V. A periosteum-derived cell line to study the role of Bmp/Tgfbeta signaling in periosteal cell behavior and function. Front Physiol (2023) 14:1221152. 10.3389/fphys.2023.1221152 37799511 PMC10547901

[95] Kumagai K Vasanji A Drazba JA Butler RS Muschler GF. Circulating cells with osteogenic potential are physiologically mobilized into the fracture healing site in the parabiotic mice model. J Orthop Res. (2008) 26(2):165–75. 10.1002/jor.20477 17729300

[96] Duchamp de Lageneste O Julien A Abou-Khalil R Frangi G Carvalho C Cagnard N Periosteum contains skeletal stem cells with high bone regenerative potential controlled by periostin. Nat Commun. (2018) 9(1):773. 10.1038/s41467-018-03124-z 29472541 PMC5823889

[97] Bonnet N Standley KN Bianchi EN Stadelmann V Foti M Conway SJ The matricellular protein periostin is required for sost inhibition and the anabolic response to mechanical loading and physical activity. J Biol Chem. (2009) 284(51):35939–50. 10.1074/jbc.M109.060335 19837663 PMC2791022

